# Interacting Environmental Stress Factors Affects Targeted Metabolomic Profiles in Stored Natural Wheat and That Inoculated with *F. graminearum*

**DOI:** 10.3390/toxins10020056

**Published:** 2018-01-29

**Authors:** Esther Garcia-Cela, Elisavet Kiaitsi, Angel Medina, Michael Sulyok, Rudolf Krska, Naresh Magan

**Affiliations:** 1 Applied Mycology Group, Environment and AgriFood Theme, Cranfield University, Cranfield MK43 0AL, UK; M.E.Garcia-Cela@cranfield.ac.uk (E.G.-C.); Elsa.Kiaitsi@cranfield.ac.uk (E.K.); a.medinavaya@cranfield.ac.uk (A.M.); 2 Center for Analytical Chemistry, Department of Agrobiotechnology (IFA-Tulln), University of Natural Resources and Life Sciences, Vienna (BOKU), Konrad Lorenzstr. 20, A-3430 Tulln, Austria; michael.sulyok@boku.ac.at (M.S.); rudolf.krska@boku.ac.at (R.K.)

**Keywords:** secondary metabolites, water activity, temperature, environmental stress, mycotoxins, *Fusarium graminearum*, storage, wheat, This is the first study to examine the effects of interacting environmental factors on secondary metabolite contamination of stored wheat grain. Colonisation by *F. graminearum* modifies the range; types and concentrations of secondary metabolites produced on stored wheat.

## Abstract

Changes in environmental stress impact on secondary metabolite (SM) production profiles. Few studies have examined targeted SM production patterns in relation to interacting environmental conditions in stored cereals. The objectives were to examine the effect of water activity (a_w_; 0.95–0.90) x temperature (10–25 °C) on SM production on naturally contaminated stored wheat and that inoculated with *Fusarium graminearum.* Samples were analysed using Liquid Chromatography-Tandem Mass Spectrometry (LC-MS/MS) on (a) total number of known SMs, (b) their concentrations and (c) changes under environmental stress. 24 *Fusarium* metabolites were quantified. Interestingly, statistical differences (ChisSq., *p* < 0.001) were observed in the number of SMs produced under different sets of interacting environmental conditions. The dominant metabolites in natural stored grain were deoxynivalenol (DON) and nivalenol (NIV) followed by a range of enniatins (A, A1, B, B1), apicidin and DON-3-glucoside at 10 °C. Increasing temperature promoted the biosynthesis of other SMs such as aurofusarin, moniliformin, zearalenone (ZEN) and their derivatives. Natural wheat + *F. graminearum* inoculation resulted in a significant increase in the number of metabolites produced (ChisSq., *p* < 0.001). For ZEN and its derivatives, more was produced under cooler storage conditions. Fusarin C was enhanced in contrast to that for the enniatin group. The relative ratios of certain groups of targeted SM changed with environmental stress. Both temperature and a_w_ affected the amounts of metabolites present, especially of DON and ZEN. This study suggests that the dominant SMs produced in stored temperate cereals are the mycotoxins for which legislation exists. However, there are changes in the ratios of key metabolites which could influence the relative contamination with individual compounds. Thus, in the future, under more extreme environmental stresses, different dominant SMs may be formed which could make present legislation out of step with the future contamination which might occur.

## 1. Introduction

In cereal commodities, it is important to ensure that drying and storage is effective to ensure that spoilage moulds and mycotoxin contamination can be prevented or minimised [1]. It is important in temperate cereals that the moisture content of 14.5–15% (wet weight basis = 0.70 water activity, a_w_) is not exceeded to ensure stable medium-term storage without spoilage and any post-harvest mycotoxin contamination. In the case of wheat/barley there are clear guidelines with regard to the EU legislative limits for contamination with type B trichothecenes (deoxynivalenol, DON) and also for Zearalenone (ZEN). However, less information is known about the range of secondary metabolites (SMs) which may be present due to fungal colonisation and whether there are differences in the relative production of different related compounds. Certainly, interacting environmental stresses of a_w_ x temperature has been shown to change the relative amounts of related DON metabolites (DON, 3- and 15-acetyl DON and Nivalenol (NIV) [2]. 

The development of both molecular and analytical techniques has resulted in significant interest in the microbiomes of different food commodities and the production of different SMs which may impact on food quality/safety. For example, recently Zhang et al. [3] examined both the fungal and bacterial microbiomes and the SM profile and changes in Pu-erh tea during fermentation. They identified the changes in bacterial and fungal communities and extrolites present and found 25 toxic metabolites, mainly of fungal origin present. Patulin and asperglaucide were the dominant SMs. For cereals, some studies have examined SM profiles and compounds in single maize kernels and stored grain [4,5]. They isolated a battery of SMs from maize kernels which suggested that the co-occurrence of different SMs may be important and have a significant impact on rural consumers in Nigeria and South Africa. A recent study on isolation of different SMs from stored wheat and correlation with molecular approaches suggested that aflatoxin B_1_, fumonisins and DON were the most common toxins present. They found a strong correlation between the presence of mycotoxin biosynthesis genes analysed by multiplex PCR and mycotoxin detection by LC/MS/MS. However, in this study, most of the wheat samples examined were stored under safe moisture conditions of <14.5% m.c. [6]. Thus, there is a lack of knowledge on what range of SMs may co-occur in wheat stored under non-conducive and conducive environmental conditions for colonisation by spoilage and mycotoxigenic fungi. 

A previous study of durum wheat inoculated with *Fusarium poae* examined the volatile organic compounds (VOCs) in inoculated and un-inoculated grain [7]. A total of 29 volatile markers were selected among the detected compounds and multivariate analysis was applied to establish the relationship between potential volatile biomarkers and fungal contamination. A range of VOCs, including alcohols, ketones, esters, furans and aromatics, were identified, both in contaminated and in healthy kernels. However, the overall volatile profile of infected samples and controls differed, indicating that the whole volatile profile, rather than individual volatile compounds, could be used to identify *F. poae* contamination of durum wheat grains [7]. However, again, this study did not examine different a_w_ x temperature storage conditions. Previously, it has been suggested that SM biosynthesis may be related to VOCs with toxigenic strains producing different biomarkers when compared to non-toxic strains in *Fusarium* section Liseola strains of *F. verticillioides* and *F. proliferatum* and also in relation to *Aspergillus* section *Nigri* species/strains [8,9]. 

The objectives of this study were to examine the effect of storage of natural wheat grain and that inoculated with *F. graminearum* stored at 0.90, 0.93, 0.95 a_w_ and 10–25 °C on (a) the range of mycotoxins and related SMs which may be produced, (b) their ranges and ratios of production. Storage conditions represented those which were non-conducive and conducive to colonisation and SM contamination. 

## 2. Results

### 2.1. Natural Fungal Contamination

**Figure 1:**
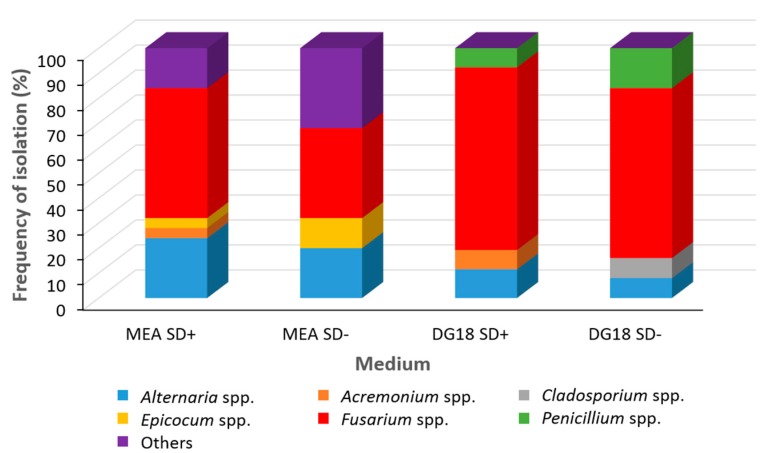
Frequency of isolation of fungi (%) from naturally contaminated harvested winter wheat on Malt Extract Agar (MEA) and Dichloran Rose Bengal 18% glycerol (DG18) media. Key: SD+ surface disinfected/ SD− non-surface disinfected.

Similar initial fungal population levels (CFUs/g dry weight) were isolated on both DG18 and MEA with Log 3.3 ± 1.3 and Log 2.4 ± 0.6 CFUs/g respectively. *Fusarium* (36–73%) was the main genus isolated, with other species isolated from the *Alternaria*, *Acremoniun*, *Cladosporium*, *Epicocum* and *Penicillium* genera (Figure 1). *Penicillium* species were only isolated from grain plated on DG18 medium. In contrast, *Epicocum* species were only isolated from wheat grain on MEA and *Acremonium* species from surface sterilized kernels.

### 2.2. Patterns of Total Targeted Metabolite Production in Relation to Interacting Environmental Factors in Naturally Stored Wheat and That Inoculated with F. graminearum

**Figure 2:**
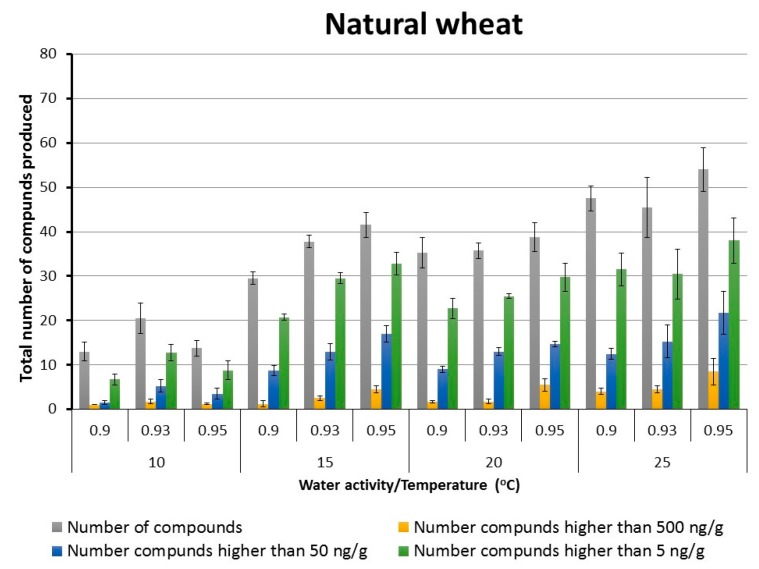
Effect of storage conditions on the total number of secondary metabolites (out of 121) produced in stored wheat grain under different interacting temperature x water activity conditions for 15 days. Data are for means + S.E.

**Figure 3:**
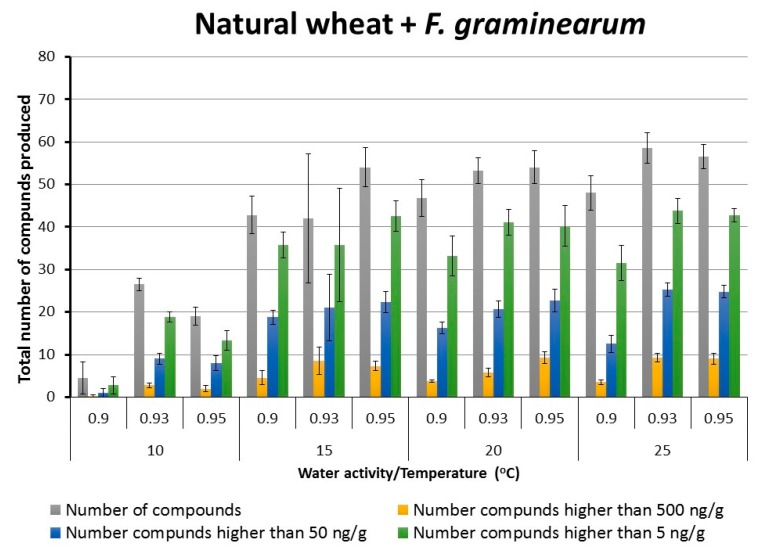
Effect of storage conditions on the total number of secondary metabolites (out of 121) produced in stored wheat grain + *F. graminearum* under different interacting temperature x water activity conditions for 15 days. Data are for means + S.E.

The naturally contaminated dry wheat grain (14.5% moisture content) used in this study contained 15 different SMs. These included DON, aurofusarin, enniatins, beauvericin and apicidin. Figure 2 and Figure 3 show the total numbers of SMs produced (out of 121) under the different storage conditions in naturally stored wheat and that with *F. graminearum*. They show the relative number of compounds produced in different categories based on the concentrations (5, 50, 500 ng/g) produced. In both treatments as temperature was increased the total number of SMs and their concentrations increased (ChiSquare, *p* < 0.05). However, in natural wheat non-parametric comparisons for each pair using the Wilcoxon method showed that the main differences in SMs produced at >5 and >50 ng/g was between 15–25 °C and that at 10 °C. a_w_ did not affect the total number of SMs produced. However, significant differences were found between 0.90–0.95 a_w_ (ChiSquare, *p* < 0.05) when examining the SM concentrations produced at >50 and >500 ng/g in both naturally stored wheat and that inoculated with *F. graminearum*.

Additionally, a linear discriminant analysis (LDA) was performed including all the variables (*p* < 0.05) (see Supplementary Figure S1). A model including 15 variables showed significant discrimination (Wilks’ Lambda < 0.0001) between natural and wheat inoculated with *F. graminareaum* (Supplementary Table S1–S4)*.* Indeed, in 92 samples only 5.4% were misclassified and all of them belonged to the inoculated group. Interestingly, for the *Fusarium* metabolites, neither ZEN nor NIV were significant. Thus, these were not included in the model. In contrast, another SM, chrysogine, could be an indicator of contamination.

Overall there were more SMs produced in stored wheat grain with additional colonization by *F. graminearum* (ChiSquare, *p* < 0.05). It was noticeable that at 15–25 °C and 0.90–0.95 a_w_ the numbers of SMs produced did not change markedly, whether considering the total number or those which were present at >50 or at >500 ng/g concentrations. These SMs were divided into those originating from colonization by *Fusarium*, *Penicillium* and *Alternaria* species in both natural and *F. graminearum* colonized wheat grain. For *Penicillium* metabolites, between 12–20 were produced, at 15–25 °C, at all the a_w_ levels examined in both naturally stored wheat and that inoculated with *F. graminearum* (Supplementary Figure S2a,b). For *Alternaria* metabolites, out of a possible 6 metabolites, at least 2–4 were often produced, regardless of environmental stress imposed (Supplementary Table S5). It is worth noting that the presence of a higher initial inoculum of *F. graminearum* (inoculation treatment) in wheat promoted the number of the SMs produced. This is indicated by the concentrations of SMs produced by *Penicillium* and *Alternaria* species. For example, altersolanol (AS) was not detected in natural wheat but was present in the wheat + *F. graminearum* treatments, especially at 0.95 a_w_ and 20–25 °C.

### 2.3. Trichothecenes, Zearalenone and Related Fusarium Metabolites in Naturally Stored Wheat and That Inoculated with F. graminearum

**Figure 4:**
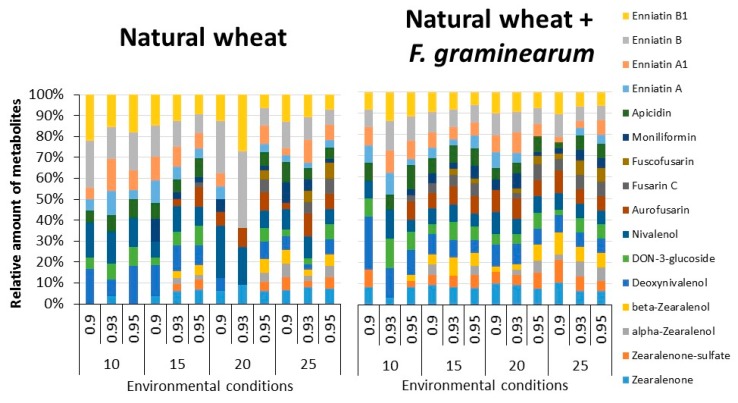
Relative production of different *Fusarium*-related secondary metabolites produced in stored naturally contaminated wheat grain and that inoculated with *F. graminearum* and stored for 15 days under different interacting temperature x water activity conditions.

Twenty-four different SMs produced by *Fusarium* were quantified in natural wheat grain and that inoculated with *F. graminearum* after 15 days storage. More *Fusarium* metabolites were produced in the stored wheat grain + *F. graminearum* treatment (ChiSquare, *p* < 0.05). Figure 4 shows the relative amounts of different *Fusarium*-related SMs isolated. Overall, between 14–16 compounds were present in the both naturally stored wheat and that inoculated with *F. graminearum*. This shows the key changes in the relative proportions of enniatins, type B trichothecenes and ZEN-related compounds found.

A breakdown of the 4 key groups of SMs found under the storage conditions for (a) ZEN-related compounds (Table 1), (b) type B trichothecenes (Table 2), (c) enniatins (Table 3) and (d) other *Fusarium* metabolites (Table 4). The Kruskal-Wallis test by ranks was used for the analysis of the effect of storage conditions and inoculation with *F. graminearum* on production of different *Fusarium*-related SMs. ZEN levels were significantly higher in stored wheat + *F. graminearum* (*p* < 0.05) inoculum treatments. For ZEN and associated compounds isolated from both types of wheat treatments, temperature significantly (*p* < 0.05) affected ZEN contamination, while a_w_ was only significant in stored wheat inoculated with *F. graminearum*. A similar trend was observed in the derivatives, alpha-zearalenone, beta-zearalenone and zearalenone-sulphate found in these stored wheat treatments (see Table 1). Temperature significantly (*p* < 0.05) affected the production of ZEN, while a_w_ was only significant in naturally contaminated stored wheat. 

The contamination profile of type B trichothecene related SMs in this study (DON and its related compounds and NIV; see Table 2) showed that both DON and NIV levels were significantly higher in stored wheat + *F. graminearum* (*p* < 0.05) inoculum. In contrast to ZEN, DON and NIV were significantly affected by a_w_ in terms of contamination levels, with optimum DON production at 15–20 °C and 0.95 a_w_. A similar trend and optimum production was observed for the DON-3-glucoside. Optimum NIV production was at the higher temperature of 25 °C.

The changes in relative amounts of enniatins produced in both stored wheat treatments are shown in Table 3. Optimum production of the four enniatins (enniatin A, A1, B, B1) was, in most cases, at 0.95 a_w_ and 15–25 °C. With *F. graminearum* inoculation there appeared to be an increase in production at 15 °C and 0.90 a_w_ which represented a significant interacting stress level. 

Apicidin, aurofusarin, fusarin C, 5-hydroxyculmorin and chrysogine concentrations were higher in the wheat + *F. graminearum* treatments in contrast to the naturally stored wheat (Table 4). Optimum production of apicidin, aurofusarin, fusarin C and for the enniatins group was at 0.95 a_w_ and 15–25 °C. A similar pattern was observed for moniliformin except at 15 °C where optimum production was at lower a_w_ levels. Optimum chrysogine production was at 0.90–0.93 a_w_.

## 3. Discussion and Conclusions

This study has focused on the effect of different steady state interacting storage environmental factors which may be conducive to *Fusarium* and other fungal colonization and the production of a range of targeted SMs produced either by the naturally contaminating mycobiota, or that inoculated with a *F. graminearum* strain. This has suggested that a wide range of different fungal species related SMs are produced depending on the temperature x water stress conditions imposed, even with a low initial level of fungal contamination. Out of a total of 121 metabolites it was noticeable that approx. 30–50 metabolites were present at 15–25 °C regardless of a_w_ conditions. At 10 °C, slower mould colonization occurs resulting in significantly less SMs being produced in both naturally contaminated stored wheat and that inoculated with *F. graminearum*. However, the relative ratio of the compounds produced in significant concentrations in these treatments, were significantly less. Thus, about 20–25 compounds were produced at >50 ng/g and <10 at >500 ng/g. This certainly suggests that, surprisingly, there are a wide range of SM compounds which are produced over a range of interacting environmental stress conditions at similar concentrations.

There have been few other studies of SM production under different storage conditions conducive to colonization by the natural mycobiota or the additional *Fusarium* inoculum. In the present study, the dry wheat grain contained ZEN, alphazol, betazol, DON, DON 3 glucoside, NIV, enniatins (4), apicidin, moniliformin, aurofusarin, fusarin C, ZEN sulphate. It was interesting that chrysogine was found to be produced in the stored grain + *F. graminearum* treatment and potentially an indicator of contamination, especially under water stress conditions. A recent study has identified the biosynthetic gene cluster for chrysogine (chry2-6) and has been found in *F. graminearum,* although predominantly present in *Penicillium* and *Aspergillus* species [10]. The production is related to nonribosomal peptide synthetases (NRPS) as deletion of these eliminated the production of chrysogine. A previous study of temperate cereals, including wheat, from different regions of Norway was carried out [11]. In wheat 20–25 compounds were found including type A (T-2; HT-2 toxin) and type B trichothecenes, ZEN and its derivatives as well as *Alternaria* toxins, enniatins, ergot alkaloids and some other *Fusarium* and *Penicillium*/*Aspergillus* SM compounds. The sample moisture contents (m.c.) were not given although they were obtained from farmers’ field in an unusually different climatic season. This suggests that the wheat grain samples at harvest may have been variable in terms of moisture content influencing the wide range of compounds isolated from some of these samples.

Other studies have mainly focused on maize and targeted profiles of SM compounds [4,5]. Indeed, Adentunji et al. [4], in an extensive survey of maize samples from different climatic regions of Nigeria, examined the production of up to 60 fungal targeted SM compounds. Besides aflatoxins and fumonisins, DON and its derivatives, *Alternaria* toxins and a range of other fungal compounds were present. The maize samples came from different storage systems, from stores to domestic environments. This would certainly influence the m.c. of the samples and perhaps fungal colonization, influencing the types and levels of contamination found. Knowledge of the actual m.c. of the samples would have been useful, as this is a useful indication of the relative quality of maize samples. Despite this, there was a correlation (negative or positive) between storage structures and level of contamination with SMs.

Fungal SM compounds are produced in four main chemical classes: polyketides, terpenoids, shikimic acid derived compounds and non-ribosomal peptides [12]. In addition, hybrid metabolites composed of moieties from different classes are common, as in the meroterpenoids, which are fusions between terpenes and polyketides. The fungi colonizing cereals are predominantly from the ascomycetes. These often have more genes of secondary metabolism than other fungal groups such as basidiomycetes, archeo-ascomycetes and chytridiomycetes. In contrast, hemi-ascomycetes and zygomycetes have none [13]. The range of metabolites found in the wheat samples colonized by field and storage fungi in the present study suggest that physiologically the dominant fungi utilize the battery of up to 16 polyketide synthases (PKS), 10 non-ribosomal protein synthases (NRPS), 2 tryptophan synthetases (TS) and 2 dimethylallyl tryptophan synthetases (DMATS) which are critically important in SM biosynthesis. Wheat represents a very nutritious finite nutrient source and this enables the *Fusarium* and other colonizing fungi to produce signature enzymes that can be enriched in secondary metabolism gene clusters and responsible for the main biosynthetic steps in SM production. Previous studies of *F. graminearum* have certainly shown that the biosynthetic gene clusters (TRI genes) responsible for SM production are directly related to the prevailing temperature x a_w_ environmental conditions. The activity of these groups of genes were correlated with these interacting environmental conditions and influenced the relative production of type B trichothecenes [14,15]. 

No ecological studies of the production of the enniatin group have been previously done. The present study suggests that in grain, optimum production of this group of compounds may occur at 20–25 °C and 0.95 a_w_. However, under sub-optimal conditions of temperature/a_w_ stress can stimulate production. This has been previously shown for other mycotoxins at a molecular level where gene clusters involved in toxin production were stimulated at sub-optimal environmental conditions [14].

Furthermore, interactions between mycotoxigenic and other spoilage fungi can also influence the relative SM production patterns. Previously, interactions between *F. culmorum* and different wheat fungal contaminants were shown to stimulate type B trichothecene production, depending on a_w_ x temperature conditions [16]. Previously, Magan and Aldred [17,18] suggested that fungal interactions and impacts on SM production are in a state of flux during storage depending on how external interacting environmental conditions naturally change and may further be influenced by the presence/absence of preservatives and the activity of storage pests. Thus, complex abiotic/biotic interactions may occur influencing the targeted SM production patterns.

This study has shown that efficiency of drying and subsequent storage will influence the relative colonisation of temperate cereals by a mixture of field (e.g., *Fusarium*, *Alternaria* species) and storage fungi (*Penicillium*, *Aspergillus* species). In Europe, *Penicillium* species, especially *P. verrucosum*, colonises grain post-harvest and results in contamination with ochratoxin A [19]. However, as environmental stress factors become more important it is possible that post-harvest mycotoxigenic fungi become more important pre-harvest and perhaps result in a significant change in the predominant SM compounds produced and perhaps also the relative ratio of related compounds [2].

## 4. Materials and Methods

### 4.1. Fungal Cultures

*F. graminearum* strain Fg 08/111 isolated from UK wheat and a known producer of ZEN and DON was used in this study. This was kindly supplied by Prof S. Edwards, Harper Adams University, Shropshire, U.K. Cultures of this strain were maintained in glycerol:water (67:33 *v/v*) at −20 °C and sub-cultured when required for experimental use.

### 4.2. Natural Fungal Contamination

#### 4.2.1. Enumeration of Fungi

Three sub-samples (10 g) were soaked for 3 h in 90 mL of sterile distilled water supplemented with 0.1% peptone (*w*/*v*) and 0.025% (*w*/*v*) Tween 80. The samples were then homogenised for 2 min in a stomacher (Lab-Blender 400: Seward Medical, London, UK). Serial dilutions (10–10^−3^). Aliquots (0.1 mL) from each dilution and for each sample were spread plated in triplicate on Dichloran 18% glycerol agar (DG18: CM0729; Oxoid) media and Malt extract agar (MEA: CM59; Oxoid) in 9 cm Petri plates and incubated at 25 °C for 7 days. The colonies growing on the plates were counted and their numbers expressed as colony forming units per gram sample (CFU/g dry weight sample).

#### 4.2.2. Fungal Identification

One hundred wheat grains were sub-sampled. Fifty kernels were first surface-disinfected with NaOCl (0.4%) for two minutes and left to dry on sterile filter paper. Subsequently, the dry kernels were directly plated (five grains per plate) on DG18 and MEA. Petri plates were incubated at 25 °C for 7 days. The plates were then inspected visually for fungal growth with the aid of a stereo microscope [20] and the percentage of fungal genera were assessed.

### 4.3. Wheat Grain Moisture Content and Water Activity Adjustment

Harvested winter wheat grain in 2016 was used in these experiments. The moisture content of the wheat grain was 14.5% and the grain was stored at 4 °C until used in experiments. Initially a moisture adsorption curve was made by adding known amounts of water to 10 g sub-samples of wheat grain which was sealed in 25 mL Universal bottles, shaken vigorously and stored overnight at 4 °C. The wheat was allowed to equilibrate at 25 °C and the water activity (a_w_; Aqualab 4TE, Decagon Devices, Pullman, WA, USA) and moisture content determined (oven drying at 105 °C for 17 h). The plot of added water against actual a_w_ was used to accurately modify the wheat grain for the storage experiments.

### 4.4. Grain Inoculation and Incubation

Natural wheat grain sub-samples (10 g) were modified to different a_w_ levels with water to 0.70, 0.90, 0.93 and 0.95 a_w_ and equilibrated as detailed previously. They were placed in 40-mL vials (Chromacol Ltd, Bath, UK) with sealable caps containing a septum for gas removal. The replicates and treatments were stored in 10 L environmental chambers which also contained glycerol-water solutions (450 mL) in a beaker to maintain the equilibrium relative humidity (ERH) of the atmosphere at the target a_w_ levels of the stored wheat samples at each experimental temperature (10, 15, 20 and 25 °C). Glycerol solutions were renewed weekly. 

Another set of replicates (*n* = 4) of the wheat grain sub-samples were inoculated with four 5-mm diameter agar disk taken from a 7 day old colony of the *F. graminearum* strain grown on V8 agar (V8^®^, 175 mL; CaCO_3_, 3g; ZnSO_4_·7H_2_O, 0.01 g; CuSO_4_·5H_2_O, 0.005 g; agar, 20 g/L) and transferred to vials containing the natural wheat grain treatments and replicates and mixed thoroughly. The storage vials were kept open in the environmental storage chambers until the end of the experiment. 

### 4.5. Secondary Metabolite Analyses

#### 4.5.1. Sample Preparation

The natural and *F. graminearum* inoculated wheat grain was dried for 48 h at 60 °C and then milled and stored at 4 °C until their analyses. Five grams of milled wheat were extracted using 20 mL extraction solvent (acetonitrile/water/acetic 79/20/1 *v*/*v*/*v*) followed by a 1 + 1 dilution using acetonitrile/water/acetic 20/79/1 *v*/*v*/*v*. Five µL of the diluted extract was directly injected into the sampling port for LC-MS/MS in the equipment for analysis.

#### 4.5.2. LC-MS/MS Parameters

LC-MS/MS screening of target fungal metabolites was performed with a QTrap 5500 LC-MS/MS System (Applied Biosystems, Foster City, CA, USA) equipped with a TurboIonSpray electrospray ionization (ESI) source and a 1290 Series HPLC System (Agilent, Waldbronn, Germany). Chromatographic separation was performed at 25 °C on a Gemini C_18_-column, 150 × 4.6 mm i.d., 5 µm particle size, preceded by a C_18_ 4 × 3 mm i.d. security guard cartridge (all from Phenomenex, Torrance, CA, USA). The chromatographic method as well as chromatographic and mass spectrometric parameters were described previously in Malachova et al. [21]. 

ESI-MS/MS was performed in the time-scheduled multiple reaction monitoring (MRM) mode both in positive and negative polarities in two separate chromatographic runs per sample by scanning two fragmentation reactions per analyte. The MRM detection window of each analyte was set to its expected retention time ± 27 s and ± 48 s in the positive and the negative mode, respectively. Confirmation of positive analyte identification was obtained by the acquisition of two MRMs per analyte (with the exception of moniliformin (MON) and 3-nitropropionic acid (3-NPA), that exhibit only one fragment ion), which yielded 4.0 identification points according to commission decision 2002/657/EC. In addition, the LC retention time and the intensity ratio of the two MRM transition agreed with the related values of an authentic standard within 0.1 min and 30% rel., respectively. Quantification was performed via external calibration using serial dilutions of a multi-analyte stock solution. Results were corrected for apparent recoveries obtained during re-validation of wheat for the extended set of analytes: zearalenone (103.9%), zearalenone-sulphate (100%), zearalenol (91.1%), deoxinivalenol (90.1%), DON-3-glucoside (55.6%), Nivalenol (60.2%), Culmorin (102.8%), 5-Hydroxyculmorin (75.5%), 15-Hydroxyculmorin (96.8%), Aurofusarin (64.4%), Fusarin C (94.2%), Fuscofusarin (100%), Moniliformin (72.4%), Butenolid (77.3%), Apicidin (106.8%), Enniatin A (115.4%), Enniatin A1 (112%), Enniatin B (110.7%), Enniatin B1 (105.8%), Enniatin B2 (101.9%), Enniatin B3 (100.8%), Chrysogine (86.3%), Gibepyron D (87.9%). 

Mycotoxins results were corrected for the recoveries. The accuracy of the method has been verified on a continuous basis by regular participation in proficiency testing schemes [21,22].

### 4.6. Statistical Analysis and Modelling the Results

Statistical analysis was performed using the package JMP^®^ Pro 13 (SAS Institute Inc., 2016. Cary, NC, USA). Datasets were tested for normality and homoscedasticity using the Shapiro-Wilk and Levene test, respectively. When data failed the normality test, variable transformation was performed to try to improve normality or homogenise the variances. Transformed data were still not normally distributed and therefore the Wilcoxon or Kruskal-Wallis test by ranks was used for the analysis of the data. Linear Discriminant Analysis (LDA) were applied to develop discrimination model covariance. Stepwise forward selection was used to choose the significant variables (*p* value < 0.05).

## Figures and Tables

**Table 1:** Production of zearalenone and related compounds in the two stored wheat grain treatments under different temperature x water activity (a_w_) conditions. Red indicates maximum production levels (ng/g) while green and shades of yellow/orange represents intermediate production levels. Key: <LOD, below limits of detection. Shading is per column.

*T* (°C)	a_w_	Natural Wheat				Natural Wheat + *F. graminearum*			
		ZEN ^1^	Alpha-ZOL ^2^	Beta-ZOL ^3^	Total Sum	ZEN ^1^	Alpha-ZOL ^2^	Beta-ZOL ^3^	Total Sum
**10**	**0.9**	<LOD	<LOD	<LOD	**0.0**	<LOD	<LOD	<LOD	**0.0**
	**0.93**	6.0	<LOD	<LOD	**6.0**	25.5	<LOD	<LOD	**25.5**
	**0.95**	<LOD	<LOD	<LOD	**0.0**	1.0	<LOD	<LOD	**1.0**
**15**	**0.9**	16.7	<LOD	<LOD	**16.7**	195.9	2.6	12.0	**210.4**
	**0.93**	18.5	1.1	3.6	**23.1**	551.8	2.7	7.4	**561.9**
	**0.95**	131.9	1.2	6.7	**139.7**	241.9	2.9	8.2	**252.9**
**20**	**0.9**	0.5	<LOD	<LOD	**0.5**	110.9	<LOD	3.2	**114.1**
	**0.93**	1.0	<LOD	<LOD	**1.0**	11.4	<LOD	<LOD	**11.4**
	**0.95**	777.5	13.3	51.9	**842.6**	810.2	9.1	35.7	**855.1**
**25**	**0.9**	112.5	2.8	16.3	**131.6**	382.6	8.5	16.4	**407.5**
	**0.93**	1536.9	19.9	84.9	**1641.7**	1489.7	13.3	81.5	**1584.5**
	**0.95**	1167.8	15.6	59.7	**1243.1**	1461.4	11.4	78.3	**1551.1**

^1^ Zearalenone, ^2^ alpha-Zearalenol, ^3^ beta-Zearalenol (µg/kg).

**Table 2:** Production of type B trichothecenes in the two stored wheat grain treatments under different temperature x water activity (a_w_) conditions. Red indicates maximum production levels (ng/g) while green and shades of yellow/orange represents intermediate production levels. Key: <LOD, below limits of detection. Shading is per column.

*T* (°C)	a_w_	Natural Wheat				Natural Wheat + *F. graminearum*			
		DON ^1^	DON-3-Glucoside ^2^	NIV ^3^	Total TCTs-B ^4^	DON ^1^	DON-3-Glucoside ^2^	NIV ^3^	Total TCTs-B ^4^
**10**	**0.9**	26.2	5.2	15.1	**46.5**	11.5	0.5	9.9	**22.0**
	**0.93**	105.8	1.2	23.9	**130.9**	112.3	4.7	31.2	**148.2**
	**0.95**	25.4	3.9	14.4	**43.7**	257.8	29.7	23.0	**310.5**
**15**	**0.9**	75.4	1.7	35.2	**112.3**	102.8	13.5	17.9	**134.1**
	**0.93**	98.7	9.0	15.7	**123.4**	1266.2	28.2	78.7	**1373.1**
	**0.95**	718.4	10.7	77.7	**806.8**	1325.1	53.5	49.2	**1427.8**
**20**	**0.9**	4.6	<LOD	15.0	**19.6**	100.2	3.3	20.1	**123.6**
	**0.93**	<LOD	<LOD	32.9	**32.9**	89.1	18.4	11.2	**118.6**
		**0.95**	806.2	33.7	34.3	**874.2**	2003.1	77.9	122.0	**2203.1**
**25**	**0.9**	113.6	5.8	8.8	**128.2**	26.5	3.2	15.5	**45.3**
	**0.93**	117.3	5.2	23.3	**145.8**	761.6	25.5	85.5	**872.5**
	**0.95**	619.6	18.8	213.8	**852.2**	1265.6	53.7	261.3	**1580.6**

^1^ Deoxynivalenol, ^2^ Deoxynivalenol-3-glucoside, ^3^ Nivalenol, ^4^ Total trichothecenes type B (µg/kg).

**Table 3:** Production of enniatins (four types and total) in the two stored wheat grain treatments under different temperature x water activity (a_w_) conditions. Red indicates maximum production levels (ng/g) while green and shades of yellow/orange represents intermediate production levels. Key: <LOD, below limits of detection. Shading is per column.

*T* (°C)	a_w_	Natural Wheat					Natural Wheat + *F. graminearum*				
		ENN A ^1^	ENN A1 ^2^	ENN B ^3^	ENN B1 ^4^	Total ENNs ^5^	ENN A ^1^	ENN A1 ^2^	ENN B ^3^	ENN B1 ^4^	Total ENNs
**10**	**0.9**	0.5	1.6	0.2	0.6	3.0	<LOD	0.6	0.6	1.6	2.9
	**0.93**	0.5	4.4	3.4	7.7	16.0	0.8	3.9	3.5	7.5	15.7
	**0.95**	10.3	32.3	5.1	21.7	69.3	<LOD	0.7	15.9	4.7	21.2
**15**	**0.9**	6.1	76.0	64.8	145.8	292.7	13.4	100.6	74.9	175.9	364.8
	**0.93**	0.6	3.9	5.6	9.4	19.4	2.0	18.4	36.0	52.9	109.3
	**0.95**	2.6	22.9	26.3	49.7	101.5	2.0	27.2	37.5	62.9	129.6
**20**	**0.9**	0.5	5.3	1.2	5.8	12.8	0.3	3.5	2.6	7.2	13.5
	**0.93**	<LOD	<LOD	0.3	0.6	0.9	0.4	4.6	16.4	19.4	40.8
	**0.95**	4.3	16.4	14.0	38.4	73.1	14.1	63.1	116.2	205.9	399.3
**25**	**0.9**	0.4	4.6	2.0	3.6	10.6	<LOD	0.2	0.2	0.4	0.9
	**0.93**	0.9	4.3	9.5	15.8	30.5	11.9	105.1	62.9	71.6	251.4
	**0.95**	38.9	174.3	125.7	304.4	643.3	2.5	37.1	185.6	177.5	402.7

^1^ Enniatin A, ^2^ Enniatin A1, ^3^ Enniatin B, ^4^ Enniatin B1, ^5^ Total Enniatins (µg/kg).

**Table 4:** Production of *Fusarium* secondary metabolites (apicidin, moniliformin, aurofusarin, fusarin C, 5-hydroxyculmorin and chrysogine) in the two stored wheat grain treatments under different temperature x water activity (a_w_) conditions. Red indicates maximum production levels (ng/g) while green and shades of yellow/orange represents intermediate production levels. Key: <LOD, below limits of detection. Shading is per column.

*T* (°C)	a_W_	Natural Wheat						Natural Wheat + *F. graminearum*					
		Apicidin	MON ^1^	Aurofusarin	Fusarin C	5-Hydroxy Culmorin	Chrysogine	Apicidin	MON ^1^	Aurofusarin	Fusarin C	5-Hydroxy Culmorin	Chrysogine
**10**	**0.9**	0.9	<LOD	<LOD	<LOD	<LOD	<LOD	1.2	<LOD	<LOD	<LOD	<LOD	7.9
	**0.93**	2.9	<LOD	<LOD	<LOD	<LOD	13.3	17.0	<LOD	<LOD	<LOD	<LOD	29.7
	**0.95**	9.5	<LOD	<LOD	<LOD	<LOD	12.4	4.7	<LOD	254.0	18.7	254.1	7.1
**15**	**0.9**	3.4	183.2	<LOD	<LOD	<LOD	7.2	12.3	16.4	628.2	38.5	<LOD	24.4
	**0.93**	13.7	18.6	914.3	<LOD	<LOD	24.9	27.1	276.6	3402.0	46.0	430.7	20.2
	**0.95**	12.2	68.5	2945.7	25.0	677.6	20.4	25.5	11.3	11,672.0	43.8	708.4	16.8
**20**	**0.9**	<LOD	2.9	91.2	<LOD	<LOD	<LOD	6.0	12.2	1343.6	<LOD	<LOD	71.4
	**0.93**	<LOD	<LOD	61.0	<LOD	<LOD	<LOD	13.2	19.7	1107.4	<LOD	<LOD	65.4
	**0.95**	45.8	5.4	9759.3	117.4	<LOD	10.5	53.9	245.3	6795.0	33.7	<LOD	46.5
**25**	**0.9**	7.6	11.5	6.3	<LOD	<LOD	14.9	4.3	<LOD	337.3	71.6	<LOD	10.5
	**0.93**	15.4	15.4	686.0	165.0	<LOD	32.5	9.9	65.7	9126.7	293.7	<LOD	24.8
	**0.95**	4.6	104.8	6912.1	797.2	<LOD	8.8	132.0	225.8	17,405.3	748.2	<LOD	24.7

^1^ Moniliformin (µg/kg).

## Supplementary Materials

The following are available online at http://www.mdpi.com/2072-6651/10/2/56/s1, Figure S1: Cannonical plot of the linear discriminant analysis between stored naturally contaminated wheat (control) and that inoculated with *F. graminearum*., Figure S2: (a) Effect of storage conditions on the *Penicillium* secondary metabolites (out of 45) produced in stored wheat grain under different interacting temperature x water activity conditions for 15 days. Data are for means + S.E., (b) Effect of storage conditions on the *Penicillium* secondary metabolites (out of 45) produced in stored wheat grain + *F. graminearum* under different interacting temperature x water activity conditions for 15 days. Data are for means + S.E. Table S1: Secondary metabolites included in the linear discriminant analysis (LDA) grouped by fungal genera, Table S2: Canonical Details calculated from the overall pooled within-group covariance matrix Wilks’ Lambda, Table S3: Discriminant scores, Table S4: Misclassified, Table S5: Production of *Alternaria* secondary metaboltes (altersolanol, altertoxin I, tentoxin, macrosporin, infectopyron, dihydroinfectoyron and total) in the two stored wheat grain treatments under different temperature x water activity conditions.

## Abbreviations

AS	altersolanol
ATX-I	altertoxin-I
DON	deoxynivalenol
ENNs	enniatins
LDA	linear discriminant analysis
MON	moniliformin
NIV	nivalenol
SMs	secondary metabolites
TEN	tentoxin
TCTs-B	trichothecenes type B
VOCs	volatile organic compounds
alpha-ZOL	alpha-Zearalenol
beta-ZOL	beta-Zearalenol
ZEN	zearalenone
