# Supplementary material for: Interacting Environmental Stress Factors Affects Targeted Metabolomic Profiles in Stored Natural Wheat and That Inoculated with F. graminearum
Source: Toxins (Basel). 2018 Jan 29;10(2):56. doi: 10.3390/toxins10020056 (PMC5848157; doi:10.3390/toxins10020056)
Supplement: Supplementary file 1 [file toxins-10-00056-s001.pdf]

Supplementary Materials: Interacting Environmental Stress Factors Affects Targeted Metabolomic Profiles in Stored Natural Wheat and That Inoculated with *F. graminearum*

Esther Garcia-Cela, Elisavet Kiaitsi, Angel Medina, Michael Sulyok, Rudolf Krska and Naresh Magan

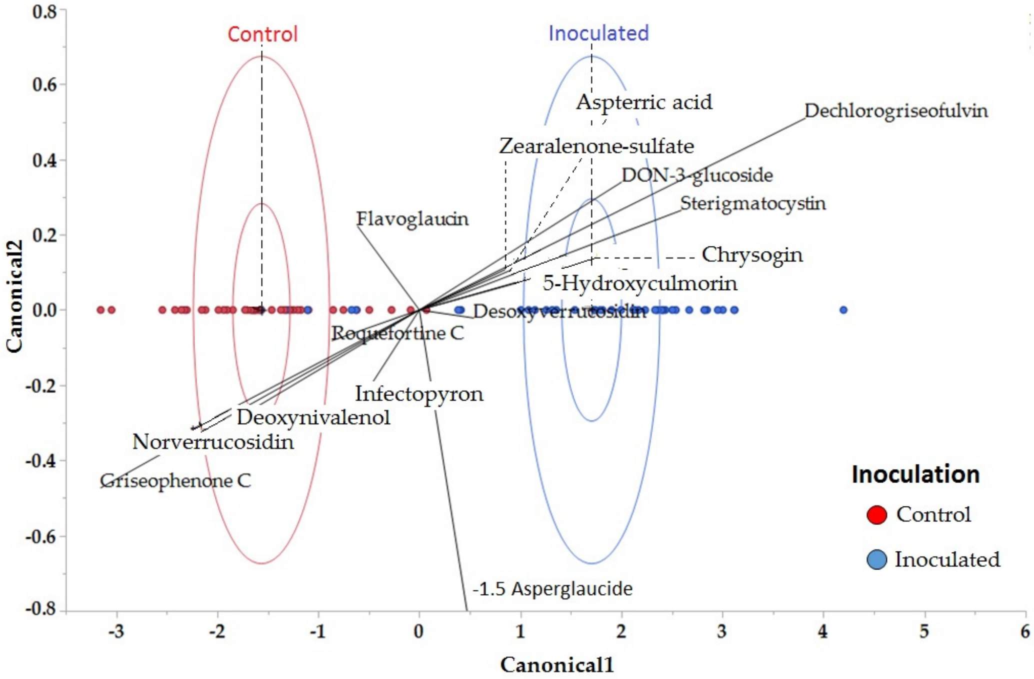

Figure S1. Cannonical plot of the linear discriminant analysis between stored naturally contaminated wheat (control) and that inoculated with *F. graminearum*.

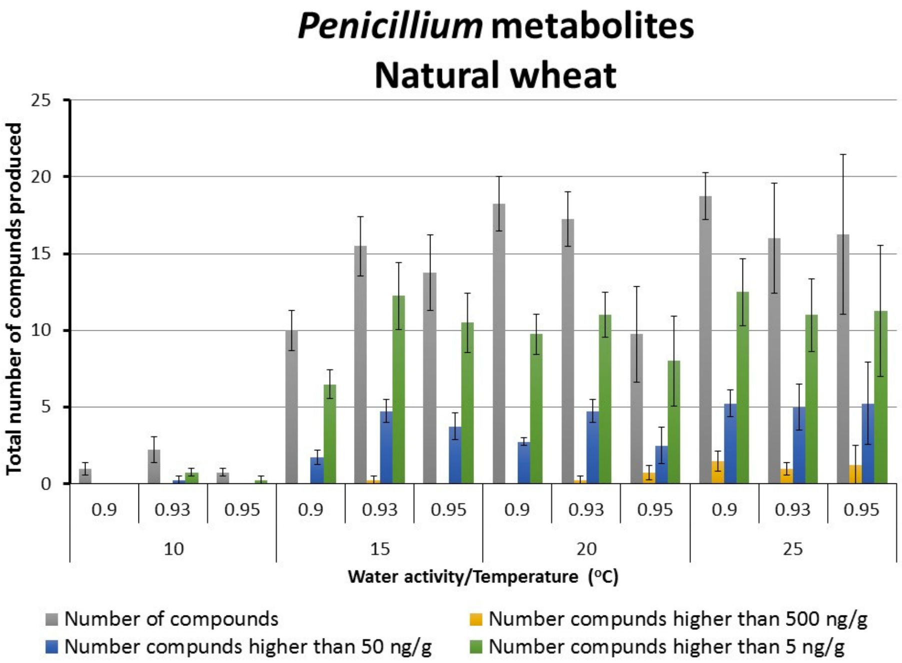

(**a**)

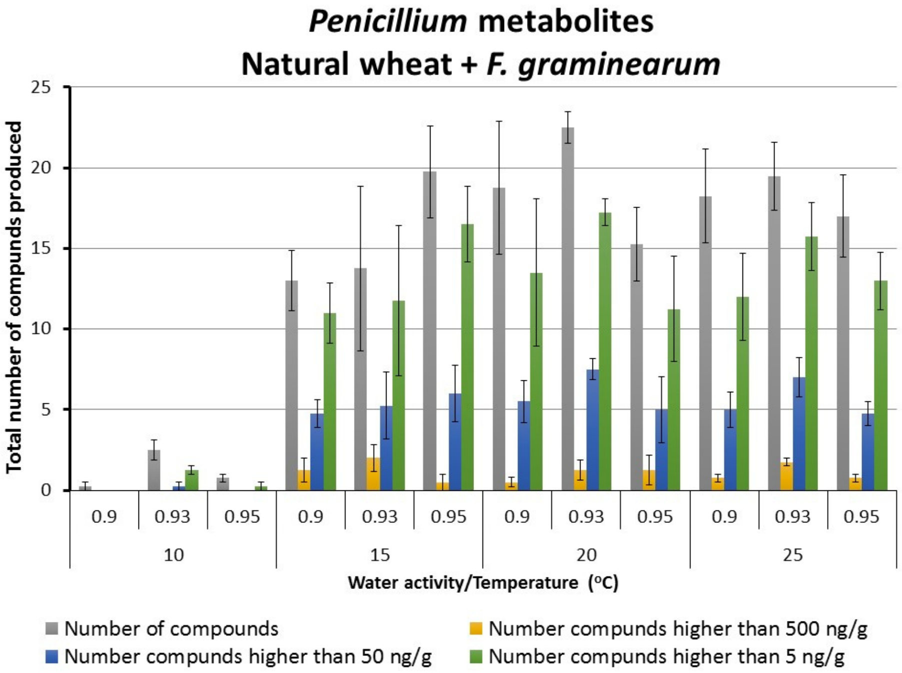

(**b**)

Figure S2. Effect of storage conditions on the *Penicillium* secondary metabolites (out of 45) produced in stored natural wheat grain (a) and in stored wheat grain + *F. graminearum* (b) under different interacting temperature x water activity conditions for 15 days. Data are for means + S.E.

Table S1. Secondary metabolites included in the linear discriminant analysis (LDA) grouped by fungal genera.

Fusarium	*Penicillium*	*Alternaria*	*Aspergillus*	Unspecific
Zearalenone-sulfate	Desoxyverrucosidin	Infectopyron	Sterigmatocystin	Asperglaucide
Deoxynivalenol	Norverrucosidin		Aspterric acid	
DON-3-glucoside	Roquefortine C			
5-Hydroxyculmorin	Griseophenone C			
Chrysogin	Dechlorogriseofulvin			
	Flavoglaucin			

Discriminant equation Canon1 = [$1.7908027 \cdot 10^{-7}$ *x* Zearalenone-sulfate + (−0.00173229067149818) *x* Deoxynivalenol + 0.0542366059471274 *x* DON-3-glucoside +0.00365544088741318 *x* 5-Hydroxyculmorin) + 0.0620752424956139 *x* Chrysogin + 0.0352002688455243 *x* Desoxyverrucosidin + −0.0370104256774014 *x* Norverrucosidin + −0.00184551544938296 *x* Roquefortine C + −0.791258703686439 *x* Griseophenone C + 0.231543704676479 *x* Dechlorogriseofulvin + (−0.00181899887872762) *x* Flavoglaucin + −0.0000759804488474338 *x* Infectopyron + 5.91681156878679 *x* Sterigmatocystin + 0.00574752284444148 *x* Aspterric acid + 0.128832906799609 *x* Asperglaucide] − 1.57772042376622.

Table S2. Canonical Details calculated from the overall pooled within-group covariance matrix Wilks' Lambda.

Eigenvalue	Percent	Cum Percent	Canonical Corr	Likelihood Ratio	Approx. F	NumDF	DenDF	Prob > F
2.73	100	100	0.86	0.268	13.81	15	76	<0.0001 *

* There are significant differences between groups.

Table S3. Discriminant scores.

Source	Count	Number Misclassified	Percent Misclassified	Entropy RSquare	−2LogLikelihood
Training	92	5	5.43	0.68	43.62

Table S4. Misclassified.

Actual	Predicted Count	
Inoculation	Control	Inoculated
Control	48	0
Inoculated	5	39

Table S5. Production of *Alternaria* secondary metaboltes (altersolanol, altertoxin I, tentoxin, macrosporin, infectopyron, dihydroinfectoyron and total) in the two stored wheat grain treatments under different temperature x water activity conditions. Red indicates maximum production levels (ng/g) while green and shades of yellow/orange represents intermediate production levels. Key: <LOD, below limits of detection. Shading is per column.

T (°C)	aw	Natural Wheat							Natural Wheat + *F. graminearum*						
		AS [1]	ATX-I [2]	TEN [3]	Macros Porin	Infecto Pyron	Dihydro Infecto Pyron	Total Sum	AS [1]	ATX-I [2]	TEN [3]	Macros Porin	Infecto Pyron	Dihydro Infecto Pyron	Total Sum
**10**	**0.9**	<LOD	<LOD	1.8	0.6	642.5	<LOD	**644.9**	<LOD	<LOD	<LOD	<LOD	507.4	<LOD	**507.4**
	**0.93**	<LOD	2.7	<LOD	<LOD	778.3	<LOD	**781.0**	<LOD	<LOD	<LOD	<LOD	582.6	<LOD	**582.6**
	**0.95**	<LOD	<LOD	<LOD	<LOD	883.0	<LOD	**883.0**	<LOD	<LOD	0.1	<LOD	860.5	<LOD	**860.7**
**15**	**0.9**	<LOD	2.3	<LOD	<LOD	547.9	<LOD	**550.1**	<LOD	4.1	<LOD	<LOD	1021.0	<LOD	**1025.0**
	**0.93**	<LOD	2.7	<LOD	<LOD	1806.5	<LOD	**1809.2**	<LOD	<LOD	<LOD	0.4	2557.0	<LOD	**2557.4**
	**0.95**	<LOD	2.6	<LOD	<LOD	5078.4	<LOD	**5081.1**	<LOD	4.4	<LOD	<LOD	3769.6	<LOD	**3773.9**
**20**	**0.9**	<LOD	<LOD	<LOD	<LOD	1228.0	<LOD	**1228.0**	194.5	1.7	0.2	8.3	3316.3	<LOD	**3521.1**
	**0.93**	<LOD	<LOD	<LOD	<LOD	1291.1	<LOD	**1291.1**	938.7	<LOD	<LOD	23.5	2188.3	<LOD	**3150.5**
	**0.95**	<LOD	7.4	<LOD	0.3	8137.1	7.4	**8152.2**	<LOD	36.6	0.2	<LOD	5757.9	6.1	**5800.8**
**25**	**0.9**	<LOD	0.8	<LOD	<LOD	1900.8	<LOD	**1901.6**	<LOD	8.6	<LOD	<LOD	3803.0	<LOD	**3811.7**
	**0.93**	<LOD	11.1	0.3	0.2	5236.9	<LOD	**5248.4**	<LOD	36.5	0.1	0.4	11,564.5	<LOD	**11,601.6**
	**0.95**	<LOD	235.6	<LOD	<LOD	19,040.3	23.5	**19,299.5**	6784.1	87.0	0.2	79.7	18,530.2	23.7	**25,505.1**

[1] Altersolanol, [2] Altertoxin-I, [3] Tentoxin (µg/kg).
